# Blood Type and Outcomes in Pregnant Women with Placenta Previa

**DOI:** 10.1155/2023/4725064

**Published:** 2023-01-25

**Authors:** Dazhi Fan, Jiaming Rao, Huishan Zhang, Dongxin Lin, Xiaoling Guo, Zhengping Liu

**Affiliations:** ^1^Foshan Institute of Fetal Medicine, Affiliated Foshan Maternity & Child Healthcare Hospital, Southern Medical University, Foshan, Guangdong 528000, China; ^2^Department of Obstetrics, Affiliated Foshan Maternity & Child Healthcare Hospital, Southern Medical University, Foshan, Guangdong 528000, China

## Abstract

**Background:**

Placenta previa increases the risks of obstetrical complications. Many studies have reported a link between various ABO blood types and pregnancy complications. This study is aimed at describing and comparing the characteristics and outcomes of women with placenta previa by ABO blood type.

**Methods:**

Data for this study was obtained from a retrospective cohort study between January 1, 2014, and June 30, 2019, of all clinically confirmed placenta previa in a university-based tertiary medical center. Both types of A, B, O, AB, and combining O and non-O blood types were compared to the characteristics and outcomes.

**Results:**

1678 participants with placenta previa were included in this study. The highest participants were blood type O with 666 (39.7%), followed by type A with 508 (30.3%) and type B with 395 (23.5%), and the lowest participants were AB with 109 (6.5%). Blood type AB had a higher incidence of antepartum hemorrhage (*p* = 0.017), predelivery anemia (*p* = 0.036), and preterm birth (*p* = 0.015) in placenta previa women. Meanwhile, the incidence of rhesus D positive (97.9% vs. 95.8%, *p* = 0.012) and twins (5.0% vs. 2.7%, *p* = 0.011) was higher in the non-O group, and the incidence of neonatal asphyxia (5.9% vs. 9.2%, *p* = 0.016) was lower in the non-O group.

**Conclusion:**

Type AB blood may be a potential risk factor for women with placenta previa. This finding may help provide any obstetrician to predict the risk of complication for placenta previa women by the ABO blood types.

## 1. Introduction

Placenta previa is defined as the placenta overlying the endocervical os and is characterized as complete, partial, marginal, and low-lying placenta depending on how much of the internal endocervical os is covered by the placenta [1, 2]. The incidence is estimated to be 5 to 12 per 1000 deliveries and varies throughout the world [3, 4]. Placenta previa is known to be associated with a marked increase in maternal and neonatal morbidity and mortality [5]. It increases the risk of peripartum hemorrhage, septicemia, prematurity, hysterectomy, and maternal and neonatal intensive care unit admission, and even maternal and fetal death [1, 6]. Previously, we have reported more than one half of placenta previa women occur antepartum hemorrhage and more than one in five has postpartum hemorrhage [7, 8]. Cesarean delivery has been confirmed as an independent risk factor for placenta previa. The other relevant risk factors included advanced maternal age, previous uterine surgery (endometrial ablation, dilatation and curettage, myomectomy, and hysteroscopic removal of intrauterine adhesions), assisted reproductive technology pregnancies, multiparity, excessive weight gain during pregnancy, and cigarette smoking [2, 9, 10].

As the first human genetic markers known, the ABO blood group system remains one of the most interesting, both clinically and scientifically, systematically dividing people into four groups [11]. Many studies have demonstrated the relationship between various ABO blood types and certain diseases including cancers, cardiovascular diseases, infections, and pregnancy complications, such as preeclampsia, gestational diabetes mellitus, and postpartum hemorrhage [11–17]. Previous studies have shown there is an association with ABO blood type and adverse pregnancy outcomes, but still, the results are conflicting. Although a case-control study found blood group O increased the risk of early-onset preeclampsia [15], studies have also reported increased preeclampsia risk with A or AB blood type [18, 19]. In a birth cohort, pregnant women with blood A type had increased risks of developing gestational diabetes mellitus [16]. Women with blood type O seemed to get more attention during pregnant because of the lower content of Factor VIII and von Willebrand factor [20]. Studies have shown blood type O to be independently associated with a risk for parturient hemorrhage [17, 21–23].

Placenta previa is direct consequence of maternal hemorrhage [1]. This leads to the premise that the characteristics and outcomes may be difference in placenta previa women among blood types. In our study, we sought to describe and compare the characteristics and outcomes of women with placenta previa by blood type in a single large university-based referral center in China. Awareness of the difference characteristics and outcomes by blood types can provide any obstetrician to predict the risk of complication for placenta previa women.

## 2. Methods

### 2.1. Participants

Data for the study was obtained from a retrospective cohort study of women with placenta previa at our hospital from January 2014 to June 2019. Approximately 13000 babies are born annually in this university-based tertiary medical center [24, 25]. All participants with placenta previa who had blood type and delivery information were included. The hospital's institutional review board approved this study (FSFY-MEC-2019-044).

### 2.2. Inclusion and Exclusion Criteria

Placenta previa was diagnosed using the last transvaginal or transabdominal ultrasonography performed before delivery; transvaginal ultrasonography was preferred if the placenta was located in the posterior wall of the uterus. The distance from the edge of the placenta to the endocervix is recorded by the trained physicians, and the placenta covers the cervical os [5]. Women whose pregnancies were terminated or who delivered before 27 w 6 d were excluded from the cohort. Both obstetrician during surgery and pathologist after cesarean delivery are all involved in the diagnosis of placenta accreta spectrum (PAS). Participants with stillbirth and sickle cell disease were excluded. Blood type is determined at the first antenatal visit based on A and B antigens on red blood cells, and further diagnosis is made before delivery by a standard blood group analysis.

### 2.3. Data Sources

Participants were identified from a prospective database of all pregnant women with a diagnosis of placenta previa that was created during the study period. The database was updated every two weeks, and there were dedicated obstetricians for maintenance and sampling inspection. Data was acquired using relevant electronic health record data including maternal characteristics (maternal age, height, and weight, gravidity, parity, previous cesarean delivery and miscarriage, fertilization way, and marital status), maternal outcomes (gestational age, blood loss, use of blood products, hemoglobin concentration, position and morphology of the placenta, and hysterectomy), and neonatal outcomes (neonatal gender, neonatal asphyxia, APGAR scores at 1^st^, 5^th^, and 10^th^ minute, and neonatal intensive care unit admission).

### 2.4. Statistical Analysis

All analyses were performed using SPSS 21.0. Placenta previa were divided into four groups based on the different blood types. We first compared the baseline characteristics and then compared outcomes among the four groups. Chi-square test was used to compare categorical variables, and *F* test was used to compare continuously distributed variables. In addition, combining O versus non-O blood types was also compared to the characteristics and outcomes. Chi-square test and an unpaired student *t* test were used for the two groups. To overcome the instability of sample size differences among the groups, we randomly selected equal sample sizes in each group for further sensitivity analysis. *P* value < 0.05 was considered statistically significant.

## 3. Results

**Figure 1:**
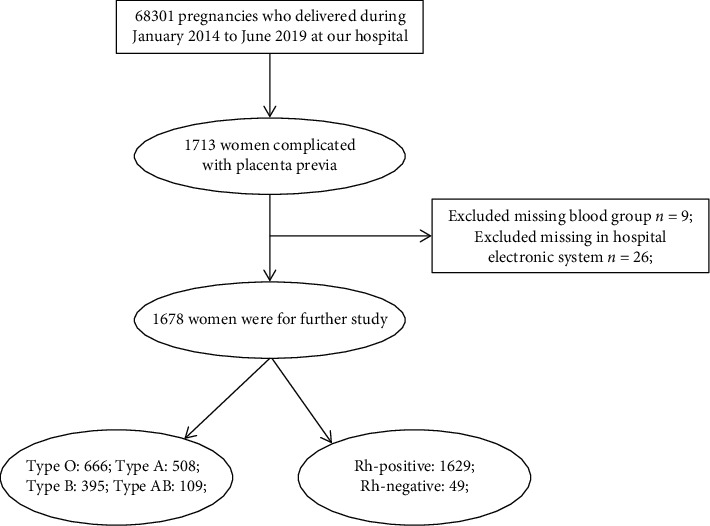
Flow chart of the participants enrolled.

There are 68301 pregnancies who delivered during the period of January 2014 to June 2019 at our hospital, and 1713 pregnancies women were diagnosed placenta previa. The prevalence of placenta previa is 25.1 per 1000 births in our data set. After excluded 35 placenta previa women, 1678 participants with placenta previa are included for further in this study (Figure 1). The highest participants are blood type O with 666 (39.7%), followed by type A with 508 (30.3%) and type B with 395 (23.5%), and the lowest participants are type AB with 109 (6.5%), and 1629 (97.1%) women have rhesus D positive and 49 (2.9%) women had rhesus D negative.

The incidence of predelivery anemia (51.0% vs. 55.7% vs. 47.6% vs. 60.0%; *p* = 0.036), antepartum hemorrhage (33.5% vs. 28.9% vs. 26.6% vs. 39.4%; *p* = 0.017), preterm birth (41.3% vs. 45.4% vs. 36.5% vs. 50.5%; *p* = 0.015), cesarean delivery (76.6% vs. 78.9% vs. 70.1% vs. 79.8%; *p* = 0.012), male newborn (56.8% vs. 61.6% vs. 53.9% vs. 65.1%; *p* = 0.044), and neonatal asphyxia (9.2% vs. 6.9% vs. 4.1% vs. 8.3%; *p* = 0.019) differs among the four groups. The incidence of predelivery anemia, antepartum hemorrhage, preterm birth, cesarean delivery, and male newborn is the highest in type AB participants, and the incidence of neonatal asphyxia is the highest in type O participants. Tables 1 and 2 show the characteristics and outcomes of the study subjects per group in greater detail. Other characteristics and outcomes are all comparable among the four groups. Sensitivity analysis shows that the results are stable (Table S1 and S2).

Tables 3 and 4 show the characteristics and outcomes between O and non-O groups. The incidence of rhesus D positive (97.9% vs. 95.8%, *p* = 0.012) and twins (5.0% vs. 2.7%, *p* = 0.011) is higher in the non-O group, and the incidence of neonatal asphyxia (5.9% vs. 9.2%, *p* = 0.016) is lower in the non-O group. Other characteristics and outcomes are also generally similar between the two groups as shown in Tables 3 and 4.

Tables 5 and 6 show the characteristics and outcomes between rhesus D positive and rhesus D negative groups. There are no significant statistical differences in characteristics and outcomes between the two groups.

## 4. Discussion

The results of this retrospective cohort indicate that placenta previa women with blood type AB are at increased risk of antepartum hemorrhage, predelivery anemia, and preterm birth when comparing other blood types. Meanwhile, blood type O may increase the risk of neonatal asphyxia in women with placenta previa.

The strength of this study is that 1678 women with placenta previa are included in a single center to analysis. To our knowledge, this may be the first study to explore the association between ABO blood types and characteristics and outcomes in placenta previa women. In addition, the results are all presented not only by the A, B, AB, and O blood types but also by combining O versus non-O blood types. The study is conducted in only a single, tertiary care center which limits the generalization of the findings to the entire population. Meanwhile, compared to A, B, and O blood types, the lowest number of participants was included in the AB blood group, and there are only 49 (2.9%) rhesus D negative-type women. Although this ratio is similar to the distribution of blood groups in the all pregnant women during this period at our hospital and the Chinese population [26, 27] and sensitivity analysis shows stability, it should be noted that this significant difference in the number of participants enrolled will skew the findings of the study. In addition, family history and race are not considered in these participants. In order to make the results of this study more complete, these factors should be covered in the following similar studies.

Placenta previa is responsible for peripartum bleeding [28]. Our previous study showed that placenta previa women have a higher risk of developing antepartum hemorrhage [7]. Studies also have demonstrated that antepartum hemorrhage could increase the risk of adverse pregnancy outcomes, such as predelivery anemia, emergency admission, preterm birth, cesarean delivery, and neonatal asphyxia [3, 29]. The rupture of the placental marginal sinus caused by the nonextension of the placenta along the cervix is considered the main cause of antepartum hemorrhage in placenta previa. The findings from our study showed that blood type AB may be a possible additional risk factor for antepartum hemorrhage. The increased risk of antepartum hemorrhage can further lead to an increased risk of other adverse pregnancy complications, such as predelivery anemia and preterm birth.

The specific mechanism of the ABO blood type and perinatal hemorrhage is still unclear. The difference in the distribution of Factor VIII and von Willebrand's Factor (vWF) in the four blood types is thought to be one of the important reasons. Study shows that the vWF plays a pivotal role in primary hemostasis through platelet subendothelial adhesion, platelet-to-platelet, and platelet aggregation [22]. In addition, it functions as a carrier of the coagulation protein Factor VIII and protects it from early degradation in the bloodstream [30]. Any quantitative or qualitative disturbance in the vWF will result in increased bleeding risk [31]. Given the large database nature of the current study, the levels of vWF are not available for analysis. Similar study may be considered to explore the vWF levels and placenta previa in the further study.

Type O blood has low levels of both the Factor VIII and vWF on the red blood cells [20, 32]. We further compared the characteristics and outcomes of type O and non-type O blood and found there was no difference in bleeding relation variables between the two groups. The incidence of rhesus D positive and multifetal gestation was higher in the non-O group, and the incidence of neonatal asphyxia was higher in the O group in this study. The mechanism behind the difference in women with placenta previa by blood type still continues to be studied.

There have been to date several studies that discussed the incidence of postpartum hemorrhage by blood type. In a single tertiary center, Drukker et al. found blood type O could increase the risk of postpartum hemorrhage [21]. Our study showed that blood type has nothing to do with postpartum hemorrhage, and this finding is in keeping with the findings of Kahr et al., Dugan-Kim et al., and Clark et al., who found there were no difference in the incidence of postpartum hemorrhage among these groups [23, 33, 34]. In a cohort of 1475 pregnant women study, Dugan-Kim et al. found that O blood type does not increase the risk of postpartum hemorrhage [33]. Kahr et al. also did not find that there was significant decrease in hemoglobin in patients with blood group O in their cohort study [23].

The discrepancies in the association of blood type with postpartum hemorrhage between different research groups may be due to the differences in race and clinical participants' characteristics. Various factors including advanced maternal age, previous history of cesarean section delivery or abortion, and smoking during pregnancy have been reported to be the risk factors of postpartum hemorrhage and placenta previa. From the findings of our study, no significant difference of such factors was observed between different blood groups. It indicated that the association between blood groups and risk of placenta previa is independent of other risk factors.

According to our study results, it was found that type AB blood may be a potential risk factor for women with placenta previa. This finding may help provide any obstetrician to predict the risk of complication for placenta previa women by the ABO blood type. For the high-risk women, we recommend that clinicians could suggest the unconfirmed placenta previa pregnancies women adjusting modifiable factors, such as weight gain during pregnancy, to reduce the incidence of placenta previa; on the other hand, targeted hemodynamic observation, possible pharmacologic prophylaxis, and care during labor and delivery can be carried out for pregnant women with confirmed placenta previa to improve the outcomes.

## Figures and Tables

**Table 1:** Maternal and gestation characteristics of the participant pregnant women by ABO blood groups.

	O (*n* = 666, 39.7%)	A (*n* = 508, 30.3%)	B (*n* = 395, 23.5%)	AB (*n* = 109, 6.5%)	*F*/*Z*/*χ*^2^ value	*p* value
Rhesus D positive (%)	638 (95.8%)	499 (98.2%)	385 (97.5%)	107 (98.2%)	6.293	0.088
Maternal age (years), mean ± SD	32.63 ± 5.18	32.75 ± 5.16	32.52 ± 5.07	32.56 ± 5.37	0.115	0.952
Advanced maternal age (>35 years old)	166 (33.7%)	131 (33.8%)	88 (32.5%)	29 (34.1%)	0.161	0.984
Maternal height (cm)	157.66 ± 5.01	157.74 ± 4.95	157.85 ± 4.81	158.60 ± 5.08	1.150	0.327
Maternal weight at delivery (kg)	65.27 ± 9.41	64.98 ± 8.40	65.22 ± 8.93	64.72 ± 9.17	0.177	0.912
Maternal BMI at delivery (kg/m^2^), mean ± SD	26.25 ± 3.50	26.09 ± 3.10	26.18 ± 3.47	25.70 ± 3.27	0.846	0.469
Married (%)	633 (95.0%)	483 (95.1%)	381 (96.5%)	105 (96.3%)	1.392	0.708
Prior miscarriages (%)	342 (51.4%)	251 (49.4%)	183 (46.3%)	62 (59.6%)	4.748	0.191
Prior cesarean delivery	235 (35.3%)	180 (35.4%)	125 (31.6%)	38 (34.9%)	1.807	0.616
Assisted reproductive techniques	62 (9.3%)	64 (12.6%)	38 (9.6%)	12 (11.0%)	3.744	0.289
Twins	18 (2.7%)	26 (5.1%)	18 (4.6%)	7 (6.4%)	6.843	0.073
Anterior placenta	206 (45.3%)	164 (45.4%)	119 (41.9%)	41 (51.3%)	2.403	0.493
Complete placenta previa	266 (39.9%)	217 (42.7%)	141 (35.7%)	46 (42.2%)	4.831	0.185
Placenta accreta spectrum	110 (16.5%)	81 (15.9%)	57 (14.4%)	17 (15.6%)	0.826	0.844
Predelivery hemoglobin (g/L)	108.00 ± 18.17	106.38 ± 17.06	109.32 ± 17.46	106.09 ± 15.12	2.356	0.070
Predelivery anemia (hemoglobin <110 g/L)	321 (51.0%)	268 (55.7%)	179 (47.6%)	63 (60.0%)	8.518	0.036
Antepartum hemorrhage	223 (33.5%)	147 (28.9%)	105 (26.6%)	43 (39.4%)	10.184	0.017

**Table 2:** Labor characteristic of the participant pregnant women by ABO blood groups.

	O (*n* = 666, 39.7%)	A (*n* = 508, 30.3%)	B (*n* = 395, 23.5%)	AB (*n* = 109, 6.5%)	*F*/*Z*/*χ*^2^ value	*p* value
Gestational age at delivery (week), mean ± SD	37.16 ± 2.47	36.86 ± 2.61	37.25 ± 2.41	36.46 ± 2.83	4.092	0.007
Preterm birth (<37 week)	270 (41.3%)	225 (45.4%)	142 (36.5%)	54 (50.5%)	10.389	0.015
Emergency admission	595 (89.3%)	442 (87.0%)	355 (89.9%)	97 (89.0%)	2.279	0.518
Cesarean delivery	510 (76.6%)	401 (78.9%)	277 (70.1%)	87 (79.8%)	10.859	0.012
Estimated blood loss (mL), median (IQR)	600 (400-1300)	600 (400-1200)	500 (350-1000)	500 (385-1000)	4.174	0.243
Postpartum hemorrhage	165 (24.8%)	123 (24.2%)	80 (20.3%)	23 (21.1%)	3.394	0.335
Cesarean hysterectomy	7 (1.1%)	8 (1.6%)	1 (0.3%)	3 (2.8%)	6.527	0.067
Transfusion of blood products	230 (34.5%)	173 (34.1%)	113 (28.6%)	37 (33.9%)	4.457	0.217
Postdelivery hemoglobin (g/L)	102.78 ± 15.42	100.95 ± 16.54	102.55 ± 14.93	101.24 ± 13.04	1.332	0.262
Postdelivery anemia (hemoglobin < 110 g/L)	390 (70.3%)	310 (71.8%)	211 (67.4%)	73 (75.3%)	2.828	0.420
Placental length (cm)	19.20 ± 2.20	19.17 ± 2.29	19.34 ± 2.59	19.03 ± 2.42	0.685	0.561
Placental width (cm)	18.65 ± 2.05	18.74 ± 2.21	18.73 ± 2.46	18.53 ± 2.35	0.388	0.762
Placental depth (cm)	2.21 ± 0.42	2.24 ± 0.44	2.21 ± 0.38	2.18 ± 0.40	0.761	0.516
Placental surface area (cm^2^)	283.65 ± 62.93	288.12 ± 82.47	288.49 ± 77.70	280.52 ± 71.88	0.713	0.544
Placental volume (cm^3^)	436.62 ± 306.50	456.01 ± 395.09	4458.42 ± 380.20	412.99 ± 160.84	0.788	0.500
Placental weight (g)	580.71 ± 178.59	585.75 ± 198.21	582.41 ± 134.38	576.11 ± 139.53	0.091	0.965
Male newborn	378 (56.8%)	313 (61.6%)	213 (53.9%)	71 (65.1%)	8.118	0.044
Neonatal asphyxia	61 (9.2%)	35 (6.9%)	16 (4.1%)	9 (8.3%)	9.931	0.019
Admission to the neonatal intensive care unit	161 (24.2%)	112 (22.0%)	77 (19.5%)	24 (22.0%)	3.170	0.367
Apgar score of <7 at 1 min	53 (8.0%)	33 (6.5%)	16 (4.1%)	9 (8.3%)	6.637	0.083
Apgar score of <7 at 5 min	5 (0.8%)	6 (1.2%)	3 (0.8%)	0 (0)	1.082	0.779
Apgar score of <7 at 10 min	1 (0.2%)	3 (0.6%)	1 (0.3%)	0 (0)	1.885	0.611

**Table 3:** Maternal and gestation characteristics of the participant pregnant women by type O vs. non-type O blood groups.

	Type O (*n* = 666, 39.7%)	Non-type O (*n* = 1012, 60.3%)	*t*/*Z*/*χ*^2^ value	*p* value
Rhesus D positive (%)	638 (95.8%)	991 (97.9%)	6.423	0.012
Maternal age (years), mean ± SD	32.63 ± 5.18	32.64 ± 5.14	0.062	0.951
Advanced maternal age (>35 years old)	166 (33.7%)	248 (33.3%)	0.015	0.902
Maternal height (cm)	157.66 ± 5.01	157.87 ± 4.91	0.871	0.384
Maternal weight at delivery (kg)	65.27 ± 9.41	65.05 ± 8.69	0.477	0.634
Maternal BMI at delivery (kg/m^2^), mean ± SD	26.25 ± 3.50	26.08 ± 3.27	0.962	0.336
Married (%)	633 (95.0%)	969 (95.8%)	0.463	0.549
Prior miscarriages (%)	342 (51.4%)	496 (49.0%)	0.879	0.369
Prior cesarean delivery	235 (35.3%)	343 (33.9%)	0.345	0.564
Assisted reproductive techniques	62 (9.3%)	114 (11.3%)	1.636	0.222
Twins	18 (2.7%)	51 (5.0%)	5.563	0.011
Anterior placenta	206 (45.3%)	324 (44.7%)	0.039	0.857
Complete placenta previa	266 (39.9%)	404 (39.9%)	0.001	0.999
Placenta accreta spectrum	110 (16.5%)	155 (15.3%)	0.435	0.538
Predelivery hemoglobin (g/L)	108.00 ± 18.17	107.49 ± 17.06	0.563	0.573
Predelivery anemia (hemoglobin < 110 g/L)	321 (51.0%)	510 (53.0%)	0.649	0.442
Antepartum hemorrhage	223 (33.5%)	295 (29.2%)	3.534	0.066

**Table 4:** Labor characteristics of the participant pregnant women by type O vs. non-type O blood groups.

	Type O (*n* = 666, 39.7%)	Non-type O (*n* = 1012, 60.3%)	*t*/*Z*/*χ*^2^ value	*p* value
Gestational age at delivery (week), mean ± SD	37.16 ± 2.47	36.97 ± 2.57	1.496	0.135
Preterm birth (<37 week)	270 (41.3%)	421 (42.4%)	0.193	0.683
Emergency admission	595 (89.3%)	894 (88.3%)	0.401	0.529
Cesarean delivery	510 (76.6%)	765 (75.6%)	0.213	0.683
Estimated blood loss (mL), median (IQR)	600 (400-1300)	500 (350-1100)	1.225	0.221
Postpartum hemorrhage	165 (24.8%)	226 (22.3%)	1.341	0.262
Cesarean hysterectomy	7 (1.1%)	12 (1.2%)	0.065	0.820
Transfusion of blood products	230 (34.5%)	323 (31.9%)	1.246	0.266
Postdelivery hemoglobin (g/L)	102.78 ± 15.42	101.58 ± 15.58	1.421	0.155
Postdelivery anemia (hemoglobin < 110 g/L)	390 (70.3%)	594 (70.5%)	0.012	0.952
Placental length (cm)	19.20 ± 2.20	19.22 ± 2.43	0.155	0.876
Placental width (cm)	18.65 ± 2.05	18.72 ± 2.32	0.587	0.557
Placental depth (cm)	2.21 ± 0.42	2.22 ± 0.42	0.538	0.591
Placental surface area (cm^2^)	283.65 ± 62.93	287.46 ± 97.51	1.083	0.279
Placental volume (cm^3^)	436.62 ± 306.50	452.34 ± 370.97	0.901	0.368
Placental weight (g)	580.71 ± 178.59	583.31 ± 169.09	0.250	0.803
Male newborn	378 (56.8%)	597 (59.0%)	0.824	0.390
Neonatal asphyxia	61 (9.2%)	60 (5.9%)	6.264	0.016
Admission to the neonatal intensive care unit	161 (24.2%)	213 (21.0%)	2.267	0.134
Apgar score of <7 at 1 min	53 (8.0%)	58 (5.7%)	3.224	0.087
Apgar score of <7 at 5 min	5 (0.8%)	9 (0.9%)	0.093	0.794
Apgar score of <7 at 10 min	1 (0.2%)	4 (0.4%)	0.812	0.654

**Table 5:** Maternal and gestation characteristics of the participant pregnant women by Rh-positive vs. Rh-negative blood groups.

	Rh-positive (*n* = 1629, 97.1%)	Rh-negative (*n* = 49, 2.9%)	*t*/*Z*/*χ*^2^ value	*p* value
Maternal age (years), mean ± SD	32.60 ± 5.15	33.81 ± 5.06	1.430	0.161
Advanced maternal age (>35 years old)	399 (24.5%)	15 (30.6%)	0.958	0.328
Maternal height (cm)	157.78 ± 4.95	158.04 ± 5.01	0.354	0.725
Maternal weight at delivery (kg)	65.07 ± 8.97	67.18 ± 9.11	1.561	0.125
Maternal BMI at delivery (kg/m^2^), mean ± SD	26.13 ± 3.35	26.86 ± 3.66	1.483	0.138
Married (%)	1557 (95.6%)	45 (91.8%)	1.542	0.214
Prior miscarriages (%)	816 (50.1%)	22 (44.9%)	0.513	0.474
Prior cesarean delivery	561 (34.4%)	17 (34.7%)	0.001	0.970
Assisted reproductive techniques	171 (10.5%)	5 (10.2%)	0.004	0.947
Twins	67 (4.1%)	2 (4.1%)	0.001	0.991
Anterior placenta	518 (45.2%)	12 (34.3%)	1.647	1.199
Complete placenta previa	653 (40.1%)	17 (34.7%)	0.577	0.448
Placenta accreta spectrum	258 (15.8%)	7 (14.3%)	0.086	0.769
Predelivery hemoglobin (g/L)	107.63 ± 17.50	109.64 ± 17.86	0.758	0.452
Predelivery anemia (hemoglobin < 110 g/L)	811 (52.5%)	20 (42.6%)	1.806	0.179
Antepartum hemorrhage	499 (30.6%)	19 (38.8%)	1.478	0.224

**Table 6:** Labor characteristics of the participant pregnant women by Rh-positive vs. Rh-negative blood groups.

	Rh-positive (*n* = 1629, 97.1%)	Rh-negative (*n* = 49, 2.9%)	*t*/*Z*/*χ*^2^ value	*p* value
Gestational age at delivery (week), mean ± SD	37.03 ± 2.54	37.37 ± 2.25	0.904	0.366
Preterm birth (<37 weeks)	674 (42.2%)	17 (36.2%)	0.676	0.411
Emergency admission	1445 (88.7%)	44 (89.8%)	0.057	0.812
Cesarean delivery	1235 (75.8%)	40 (81.6%)	0.883	0.347
Estimated blood loss (mL), median (IQR)	500 (380-1200)	500 (350-800)	1.262	0.207
Postpartum hemorrhage	382 (23.4%)	9 (18.4%)	0.688	0.407
Cesarean hysterectomy	18 (1.1%)	1 (2.0%)	0.372	0.542
Transfusion of blood products	543 (33.3%)	10 (20.4%)	3.597	0.058
Postdelivery hemoglobin (g/L)	102.01 ± 15.56	103.31 ± 14.49	0.551	0.581
Postdelivery anemia (hemoglobin < 110 g/L)	952 (70.4%)	32 (71.1%)	0.010	0.920
Placental length (cm)	19.21 ± 2.34	19.35 ± 2.14	0.409	0.682
Placental width (cm)	18.67 ± 2.21	19.33 ± 2.37	2.033	0.062
Placental depth (cm)	2.22 ± 0.42	2.19 ± 0.41	0.386	0.701
Placental surface area (cm^2^)	285.62 ± 73.55	296.74 ± 68.16	1.122	0.267
Placental volume (cm^3^)	443.06 ± 322.98	545.94 ± 300.76	0.894	0.376
Placental weight (g)	581.18 ± 174.13	614.27 ± 130.90	1.494	0.143
Male newborn	950 (58.3%)	25 (51.0%)	1.041	0.308
Neonatal asphyxia	120 (9.2%)	1 (5.9%)	2.016	0.156
Admission to the neonatal intensive care unit	365 (22.4%)	9 (18.4%)	0.448	0.503
Apgar score of <7 at 1 min	110 (6.8%)	1 (2.0%)	1.710	0.191
Apgar score of <7 at 5 min	14 (0.9%)	0 (0.0%)	0.425	0.999
Apgar score of <7 at 10 min	5 (0.3%)	0 (0.0%)	0.151	0.999

## Data Availability

The datasets used and/or analyzed during the current study are available from the corresponding authors on reasonable request.

## Supplementary Materials

Supplementary Materials Table S1: maternal and gestation characteristics of the participant pregnant women by ABO blood groups (sensitivity analysis). Table S2: labor characteristic of the participant pregnant women by ABO blood groups (sensitivity analysis).
