# Supplementary material for: Blood Type and Outcomes in Pregnant Women with Placenta Previa
Source: Oxid Med Cell Longev. 2023 Jan 25;2023:4725064. doi: 10.1155/2023/4725064 (PMC9891818; doi:10.1155/2023/4725064)
Supplement: Supplementary Materials — Table S1: maternal and gestation characteristics of the participant pregnant women by ABO blood groups (sensitivity analysis). Table S2: labor characteristic of the participant pregnant women by ABO blood groups (sensitivity analysis). [file 4725064.f1.docx]

Table S1. Maternal and gestation characteristics of the participant pregnant women by ABO blood groups (sensitivity analysis).

|  | O (n=100) | A (n=100) | B (n=100) | AB (n=100) | F/Z/χ^2^ value | *P* value |
| --- | --- | --- | --- | --- | --- | --- |
| Rhesus D positive (%) | 93 (93.0%) | 97 (97.0%) | 99 (99.0%) | 98 (98.0%) | 5.511 | 0.155 |
| Maternal age (years), mean±sd | 32.18±4.97 | 32.67±4.94 | 32.87±5.06 | 32.57±5.50 | 0.244 | 0.866 |
| Advanced maternal age (>35 years old) | 24 (24.0%) | 23 (23.0%) | 26 (26.0%) | 27 (27.0%) | 0.685 | 0.876 |
| Maternal height (cm) | 157.71±5.21 | 156.86±5.21 | 157.44±4.42 | 158.31±5.06 | 1.428 | 0.234 |
| Maternal weight at delivery (kg) | 64.36±7.97 | 64.47±7.86 | 65.00±7.97 | 64.67±8.29 | 0.128 | 0.944 |
| Maternal BMI at delivery (kg/m^2^), mean±sd | 25.82±2.79 | 26.12±2.71 | 26.24±3.36 | 25.85±3.27 | 0.429 | 0.733 |
| Married (%) | 94 (94.0%) | 92 (92.0%) | 95 (95.0%) | 97 (97.0%) | 2.501 | 0.502 |
| Prior miscarriages (%) | 51 (51.0%) | 59 (59.0%) | 47 (47.0%) | 59 (59.0%) | 4.348 | 0.232 |
| Prior cesarean delivery | 34 (34.0%) | 34 (34.0%) | 29 (29.0%) | 36 (36.0%) | 1.205 | 0.763 |
| Assisted reproductive techniques | 4 (4.0%) | 12 (12.0%) | 11 (11.0%) | 9 (9.0%) | 4.640 | 0.220 |
| Twins | 2 (2.0%) | 5 (5.0%) | 3 (3.0%) | 6 (6.0%) | 2.539 | 0.522 |
| Anterior placenta | 38 (38.0%) | 36 (36.0%) | 26 (26.0%) | 38 (38.0%) | 5.429 | 0.144 |
| Complete placenta previa | 41 (41.0%) | 41 (41.0%) | 40 (40.0%) | 43 (43.0%) | 0.196 | 0.984 |
| Placenta accreta spectrum | 22 (22.0%) | 13 (13.0%) | 18 (18.0%) | 17 (17.0%) | 2.840 | 0.454 |
| Predelivery hemoglobin (g/L) | 104.63±16.82 | 106.34±17.12 | 110.72±17.87 | 105.88±15.21 | 2.392 | 0.068 |
| Predelivery anemia (hemoglobin < 110 g/L) | 51 (54.0%) | 52 (52.0%) | 41 (41.0%) | 62 (62.0%) | 8.848 | 0.032 |
| Antepartum hemorrhage | 35 (35.0%) | 25 (25.0%) | 26 (26.0%) | 42 (42.0%) | 8.915 | 0.033 |

Table S2. Labor characteristic of the participant pregnant women by ABO blood groups (sensitivity analysis).

|  | O (n=100) | A (n=100) | B (n=100) | AB (n=100) | F/Z/χ^2^ value | *P* value |
| --- | --- | --- | --- | --- | --- | --- |
| Gestational age at delivery (week), mean±sd | 37.19±2.42 | 36.69±2.67 | 37.36±2.30 | 36.13±2.89 | 5.246 | 0.023 |
| Preterm birth (<37 week) | 45 (45.0%) | 47 (47.0%) | 32 (32.0%) | 55 (55.0%) | 11.032 | 0.012 |
| Emergency admission | 89 (89.0%) | 83 (83.0%) | 92 (92.0%) | 89 (89.0%) | 4.123 | 0.271 |
| Cesarean delivery | 79 (79.0%) | 74 (74.0%) | 62 (62.0%) | 81 (81.0%) | 11.331 | 0.010 |
| Estimated blood loss (mL), median (IQR) | 600 (350-1550) | 600 (400-1500) | 600 (400-1200) | 500 (400-1000) | 0.998 | 0.802 |
| Postpartum hemorrhage | 27 (27.0%) | 28 (28.0%) | 27 (27.0%) | 22 (22.0%) | 1.143 | 0.781 |
| Cesarean hysterectomy | 1 (1.0%) | 2 (2.0%) | 0 (0.0%) | 3 (3.0%) | 3.154 | 0.525 |
| Transfusion of blood products | 41 (41.0%) | 40 (40.0%) | 29 (29.0%) | 35 (35.0%) | 3.927 | 0.277 |
| Postdelivery hemoglobin (g/L) | 101.97±14.48 | 101.15±15.36 | 102.21±15.10 | 101.06±13.37 | 1.140 | 0.936 |
| Postdelivery anemia (hemoglobin < 110 g/L) | 66 (66.0%) | 63 (63.0%) | 81 (81.0%) | 67 (67.0%) | 2..240 | 0.630 |
| Placental length (cm) | 19.19±2.21 | 18.76±2.14 | 19.23±2.00 | 18.98±2.46 | 0.968 | 0.408 |
| Placental width (cm) | 18.54±2.16 | 18.49±2.45 | 18.59±2.54 | 18.45±2.30 | 0.065 | 0.979 |
| Placental depth (cm) | 2.16±0.37 | 2.25±0.53 | 2.16±0.37 | 2.17±0.40 | 0.966 | 0.409 |
| Placental surface area (cm^2^) | 282.01±64.00 | 275.56±65.35 | 282.89±60.85 | 278.61±72.02 | 0.256 | 0.857 |
| Placental volume (cm^3^) | 407.92±124.41 | 413.23±142.21 | 472.80±552.83 | 409.64±163.05 | 1.048 | 0.371 |
| Placental weight (g) | 579.92±113.46 | 608.85±184.80 | 591.01±147.61 | 579.92±113.46 | 0.610 | 0.818 |
| Male newborn | 60 (60.0%) | 56 (56.0%) | 47 (47.0%) | 67 (67.0%) | 8.552 | 0.038 |
| Neonatal asphyxia | 11 (11.0%) | 8 (8.0%) | 5 (5.0%) | 9 (9.0%) | 2.477 | 0.518 |
| Admission to the neonatal intensive care unit | 26 (26.0%) | 27 (27.0%) | 16 (16.0%) | 23 (23.0%) | 4.178 | 0.254 |
| Apgar score of <7 at 1 min | 12 (12.0%) | 8 (8.0%) | 5 (5.0%) | 8 (8.0%) | 3.270 | 0.377 |
| Apgar score of <7 at 5 min | 1 (1.0%) | 1 (1.0%) | 1 (1.0%) | 0 (0.0) | 1.008 | 0.999 |
| Apgar score of <7 at 10 min | 0 (0.0%) | 1 (1.0%) | 0 (0.0%) | 0 (0.0) | 3.008 | 0.999 |
